# Doxorubicin-induced cardiotoxicity: Is ferroptosis the primary driver or a downstream amplifier?

**DOI:** 10.17179/excli2026-9516

**Published:** 2026-07-02

**Authors:** Yogender Goswami, Nisha Sharma, Umashanker Navik

**Affiliations:** 1 Department of Pharmacology, Central University of Punjab, Bathinda, Punjab 151401, India; 2 Department of Pharmacology, M. M. College of Pharmacy, Maharishi Markandeshwar (Deemed to be University), Mullana, Ambala, Haryana 133207, India

**Keywords:** doxorubicin, cardiotoxicity, ferroptosis, oxidative stress, lipid peroxidation

## Abstract

Doxorubicin (Dox) is one of the most effective anticancer agents used to treat a wide range of solid tumors as well as hematological malignancies. However, its associated cardiotoxicity poses a major challenge for its therapeutic use. There are numerous studies exploring the underlying cellular mechanisms behind Dox-induced cardiotoxicity. Apart from the well-established apoptosis and necrosis pathways, ferroptosis is a recently identified regulated cell death pathway being studied in the context of drug-induced cardiotoxicity. Under normal physiology, cardiomyocytes maintain a highly regulated iron homeostasis, while the polyunsaturated fatty acid-rich membrane also renders it susceptible to peroxidation, a hallmark of ferroptosis. Dox-induced cardiotoxicity disrupts the coordinated control of iron metabolism, generating reactive oxygen species, propagating lipid peroxidation, and impairing mitochondrial function. Progressive structural damage and functional loss of cardiomyocytes culminate in permanent cardiac cell death. Therefore, targeting regulatory nodes of ferroptosis may be beneficial for ameliorating Dox-induced cytotoxicity. However, it is still not clear whether the ferroptotic process merely acts as an initiator or can further act as an amplifier to upregulate the downstream signaling molecules in this cell death cascade. This review offers an overview of perspectives on the ferroptotic pathway and introduces readers to a novel driver-amplifier concept.

See also the graphical abstract(Fig. 1).

## 1. Introduction

Doxorubicin (Dox) is a member of the anthracycline family, which is isolated as a secondary metabolite from a mutant strain of *Streptomyces* *peucetius* (Minotti et al., 2004[46]). Dox remains one of the most effective cytotoxic drugs, used either alone or in combination with other anticancer agents (Rivankar, 2014[60]). The clinical utility of nearly all conventional anticancer drugs is restricted by significant organ toxicity, predominantly in the case of Dox. Despite its extensive therapeutic use, cardiotoxicity remains a major obstacle to its clinical application (Rawat et al., 2021[59]). This well-known toxicity depends on the cumulative dose (with risk increasing above a level of 700 mg/m^2^) and may manifest years after treatment, leading to poor outcomes (Jain, 2000[34]). Several investigations have been conducted to elucidate the cellular pathogenesis of Dox-induced cardiotoxicity. Multiple regulated cell death pathways, including apoptosis (Zhao and Zhang, 2017[92]), necroptosis (Yu et al., 2020[86]), pyroptosis (Ping et al., 2023[56]), and ferroptosis (Bhadra et al., 2025[6]), have been identified in anthracycline cardiotoxicity; however, their hierarchical relationships remain unclear. Among these, ferroptosis has been observed as a potential mechanism that accelerates cardiomyocyte death.

Ferroptosis is distinguished from other regulated cell death pathways by its dependence on iron and its characteristic accumulation of lipid peroxidation (Dixon et al., 2012[17]). Morphologically, this type of cell death is characterized by a significantly increased mitochondrial membrane density, resulting in the distortion of mitochondrial cristae and ultimately leading to mitochondrial shrinkage (Ichikawa et al., 2014[33]). Recent studies increasingly recognize the position of ferroptosis in the progression of Dox-induced cardiotoxicity (Bhadra et al., 2025[6]; Han et al., 2026[29]; Wu et al., 2024[78]). However, its exact position in the cardiotoxic cascade is still unclear. The major focus of this review is to explore the driver-amplifier concept underlying the pathogenesis of Dox-induced ferroptosis-mediated cardiotoxicity. In Dox-mediated cytotoxicity, multiple cell death pathways are simultaneously triggered, making it difficult to pinpoint which cellular pathway is responsible for initiating cell death (driver) or which one is merely involved in the progression of the event (amplifier). The driver mechanism is typically activated early in the cascade, directly triggering some of the biomolecules involved in regulated cell death. While the amplifier mechanism does not always initiate cell death, it can accelerate the process through the activation of downstream stress signaling molecules or *via *a positive feedback loop involving oxidative stress, mitochondrial dysfunction, or loss of cell membrane integrity. Determining the exact role of an event is crucial for therapeutic application, as targeting the driver pathway prevents the initiation of the cascade, whereas targeting an amplifier can slow down progression. In this context, ferroptosis emerges as a unique cellular event that, when directly causing lipid membrane destruction, can act as a driver. Alternatively, if lipid peroxidation occurs secondary to some ongoing cellular damage, ferroptosis may function as an amplifier in the cytotoxicity cascade.

This review aims to discuss the unanswered questions about the mechanism of ferroptosis-mediated cardiotoxicity. We have explained the driver-versus-amplifier concept in regulated cell death pathways and attempted to place ferroptosis within this framework.

## 2. Overview of Ferroptosis

**Figure 2:**
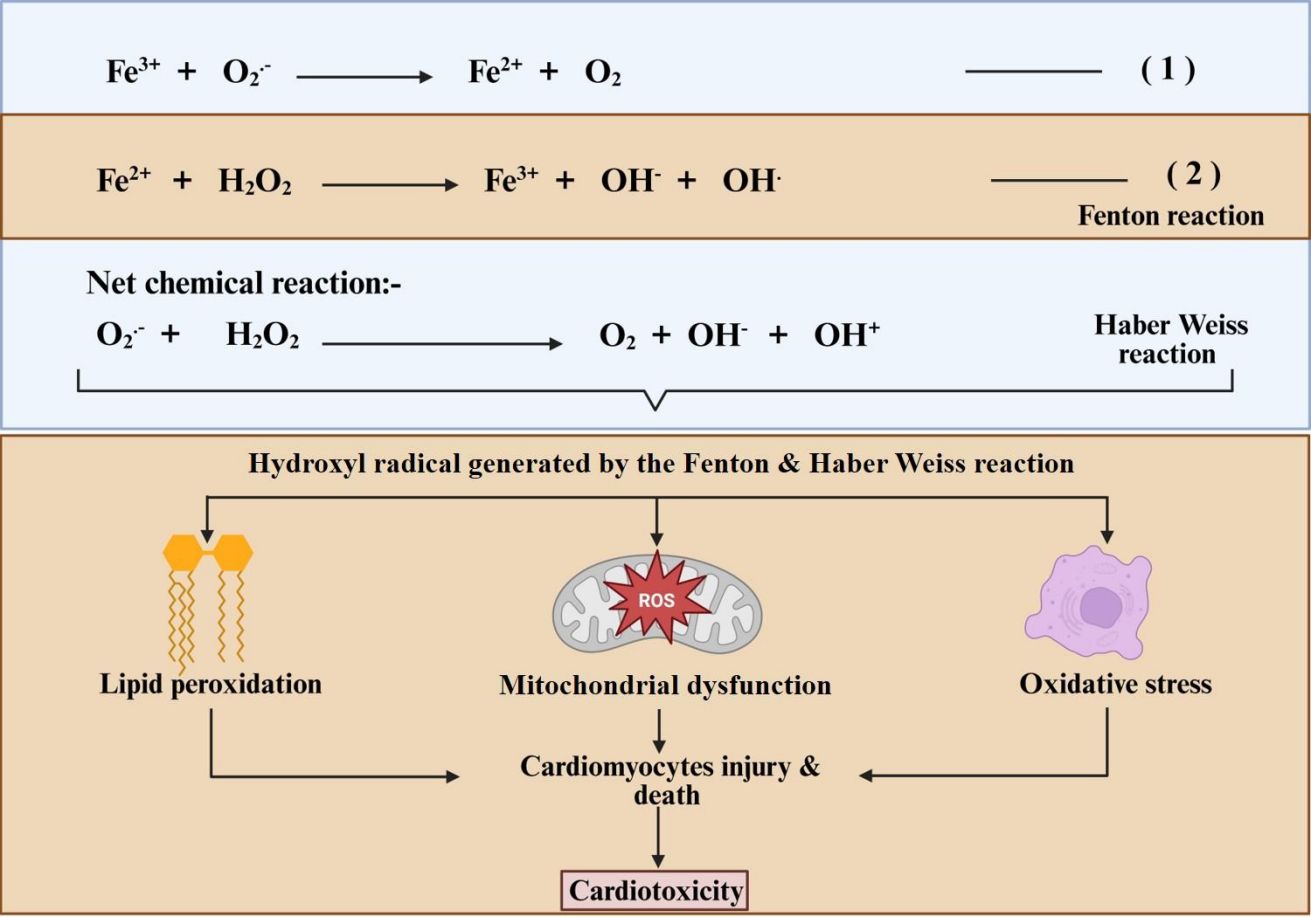
Fenton reaction and its contribution to oxidative stress in doxorubicin-induced cardiotoxicity. Doxorubicin enhances intracellular iron accumulation and redox cycling, promoting hydroxyl radical generation. This results in excessive ROS production, lipid peroxidation, mitochondrial impairment, and ultimately cardiomyocyte injury. The figure was created using BioRender software.

Ferroptosis, earlier referred to as oxytosis, is basically an iron-dependent regulated cell death that is distinguished by the buildup of cellular lipid peroxides (Dixon and Olzmann, 2024[18]). It differs from other types of programmed cell death in terms of biochemistry, genetics, and morphology (Dixon and Olzmann, 2024[18]). Although various studies suggest different mechanisms for its initiation, disruption of the glutathione-dependent antioxidant system primarily triggers ferroptosis, leading to uncontrolled lipid peroxidation (Li et al., 2022[40]). Due to the reduced intracellular glutathione (GSH) and glutathione peroxidase 4 (GPX4) levels, the accumulated lipid peroxides cannot be broken down, contributing to the formation of reactive oxygen species (ROS) (Li et al., 2022[40]). Furthermore, the presence of intracellular Fe^2+ ^ions accelerates this process by oxidizing lipids *via* Fenton reaction, which stimulates the process of ferroptosis (Figure 2(Fig. 2)) (Tang et al., 2000[69]). The only known cellular iron exporter, SLC40A1, which encodes ferroportin, the primary intracellular iron storage protein ferritin heavy chain 1 (FTH1), and the receptor for cellular iron uptake, transferrin receptor (TFRC), is a key regulator affecting cellular susceptibility to ferroptosis (Chen et al., 2020[11]). Ferroptosis susceptibility is mostly determined by the balance between iron import and export. SLC40A1 is located in the plasma membrane, which exports intracellular Fe^2+ ^to the extracellular space, thus limiting the amount of substrate needed for the ferroptotic process (Donovan et al., 2005[19]). Therefore, in case of low expression of SLC40A1, there's increased susceptibility to ferroptosis because of intracellular iron accumulation. Hepcidin, a peptide hormone, binds to SLC40A1, causing its internalization and degradation (Nemeth and Ganz, 2021[51]).

In contrast, TFRC serves as the central pathway for cellular iron uptake, which is subsequently reduced and then released into the cytosolic iron pool (Gammella et al., 2017[25]). Ferritin is a heteropolymeric molecule made up of heavy (FTH1) and light (FTL) chains (Arosio et al., 2017[2]). Having ferroxidase activity, FTH1converts the redox-active Fe^2+^ ions into less reactive Fe^3+ ^ions, permitting these ions to be accumulated easily (Arosio et al., 2017[2]). FTH1 directly restricts the supply of labile iron required for lipid peroxidation. Any abnormality in the expression of FTH1 can affect the export or storage of labile iron, causing excess iron to build up within mitochondria, where it ultimately catalyzes the synthesis of ROS, stimulating mitochondrial lipid peroxidation. This mitochondrial involvement is evident in the distinctive microscopic changes observed during ferroptosis, including decreased mitochondrial size, increased membrane density, and loss of cristae integrity (Wang et al., 2020[76]).

In mitochondrial ferroptosis, polyunsaturated fatty acids (PUFAs), especially those esterified into phospholipids, are essential substrates for lipid peroxidation (Wang et al., 2020[76]). Mostly PUFAs, such as arachidonic acid and adrenic acid, have several double bonds separated by methylene groups, providing bis-allylic hydrogen sites that are highly susceptible to oxidation (Mortensen et al., 2023[48]). Because of the peculiar enrichment of PUFA-containing phospholipids in the cardiac membranes, the heart is particularly vulnerable to lipid peroxidation and, as a result, ferroptosis (Cho et al., 2025[12]). The strong metabolic and oxidative demands of the heart further increase this susceptibility in cardiac cells (Fang et al., 2023[21]). This ROS production rises sharply in pathological conditions like cardiomyopathy, cardiac ischemia, heart failure, or any drug-induced cardiotoxicity (Fang et al., 2023[21]).

## 3. Early Events in Doxorubicin-Induced Cardiotoxicity

Dox-induced cardiotoxicity has been connected with numerous pathological events, including increased oxidative stress (Songbo et al., 2019[64]), disruption of mitochondrial function (Green and Leeuwenburgh, 2002[28]), inhibition of topoisomerase II β (Lyu et al., 2007[45]), iron dysregulation (Gammella et al., 2014[26]), and calcium overload (Shinlapawittayatorn et al., 2022[62]). These events consequently lead to cardiac cell death through various pathways, including autophagy, apoptosis, necrosis, or, more recently studied, ferroptosis. The disruption of redox homeostasis in cardiac cells is considered one of the primary molecular events initiated by Dox (Cappetta et al., 2017[8]). The quinone moiety of Dox is reduced by mitochondrial complex I to release a semiquinone radical, which then combines with molecular oxygen to produce superoxide anions (Cappetta et al., 2017[8]). ROS are immediately produced as a result, even before obvious cellular damage is evident. Early changes in iron-regulatory proteins occur concurrently with the inhibition of SLC40A1, leading to an increased accumulation of intracellular iron (Gammella et al., 2014[26]). Furthermore, the labile iron pool (LIP) is increased by ferritinophagy and an increase in the activity of the transferrin receptor, which releases stored iron into the cytosol (Zhou et al., 2022[93]). This accumulation of intracellular iron is a crucial early event that renders cardiac cells more susceptible to oxidative damage.

Given the high mitochondrial density in cardiac cells, the mitochondria are a distinctive early target of Dox (Wallace et al., 2020[73]). Following its binding to cardiolipin, Dox disrupts the electron transport chain (ETC), resulting in sequestration and electron leakage, which further increases ROS production. All mitochondrial-damaging events are simultaneously triggered, including loss of membrane potential, structural abnormalities of the cristae, loss of integrity, and, ultimately, the release of cytochrome c and pro-apoptotic factors that mediate apoptosis (Wallace et al., 2020[73]). By interfering with the ETC and decreasing ATP synthesis, Dox damages mitochondrial DNA (mtDNA), resulting in significant energy deprivation that is especially stressful for high-energy-demanding cells like cardiomyocytes.

Lipid peroxidation, especially in the PUFA-rich cardiomyocytes, is another crucial early event. Lipid hydroperoxides build up when antioxidant defenses, particularly the GSH-GPX4 axis, are compromised. Additionally, Dox causes topoisomerase II β-mediated DNA damage and transcriptional dysregulation affecting mitochondrial biogenesis and antioxidant defence (Lyu et al., 2007[45]). Fundamentally, cardiac cells are particularly susceptible to this damage because they express Top2β rather than Top2α (Vejpongsa and Yeh, 2014[71]). Studies have also focused on the effect of Dox on calcium homeostasis in relation to the heart contractile dysfunction (Shinlapawittayatorn et al., 2022[62]). Cellular vital calcium channels and pumps, such as the sodium-calcium exchanger (NCX) and the sarcoplasmic endoplasmic reticulum calcium ATPase (SERCA), are known to be strongly inhibited by Dox (Zhang et al., 2014[90]). This inhibition increases the intracellular calcium concentration. Raised Ca^2+ ^levels further incite the opening of the mitochondrial permeability transition pore, priming the cell for irreversible damage. These irreversible cellular damages often precede functional declines (Montaigne et al., 2011[47]). It has been demonstrated that long-term changes in calcium homeostasis raise the risk of arrhythmias (Deo et al., 2017[15]). Reduced contractility has been observed in Dox-treated cells due to alterations in ryanodine receptors and phospholamban, which affect the excitation-contraction coupling process (Zhang et al., 2014[90]). Clinical signs of Dox-induced cardiotoxicity may appear later in chemotherapy and within a year of therapy completion. Within days, acute early cardiotoxicity may manifest as left-ventricular dysfunction or slight ECG abnormalities (Kumar and Tiwari, 2025[37]). Progressive cardiomyocyte damage that results in cardiac failure is a hallmark of chronic, delayed cardiotoxicity (Octavia et al., 2012[53]).

## 4. Role of Ferroptosis in Different Stages of Cardiotoxicity

The pathogenesis of Dox-induced cardiotoxicity is still a topic of discussion, and its pathogenesis remains controversial and incompletely defined.

### 4.1 Early stage: Initiation and metabolic imbalance

Dox initiates ferroptosis by targeting key regulatory genes involved in iron metabolism and redox homeostasis. This theory is based on the fact that there's a significant restitution of iron stores following treatment by dexrazoxane, an FDA-approved drug for Dox-induced cardiotoxicity. Through coordinated regulation of uptake (via the transferrin receptor), storage (via ferritin), and export (via SLC40A1), intracellular iron is tightly controlled under physiological conditions (Zhang et al., 2024[91]). This balance is disrupted by Dox on several levels, deactivating iron regulatory proteins that control ferritin and transferrin receptor expression, causing the accumulation and steady increase of redox-active iron (Qin et al., 2021[57]). Dox and Fe^3+ ^can combine to form a Dox-Fe^3+ ^complex, which further gets converted into a Dox-Fe^2+ ^complex. This complex further leads to the formation of highly reactive hydroxyl radicals or highly toxic iron-peroxo complexes, consequently disrupting the DNA. Additionally, Dox reduces MITOL, further decreasing mitochondrial GPX4 expression and increasing Chac1-dependent GSH breakdown (Kitakata et al., 2021[36]). This condition allows lipid peroxides to accumulate beyond a threshold level in the presence of abundant iron and ROS, leading to permanent membrane damage and the initiation of ferroptosis.

### 4.2 Intermediate stage: Lipid peroxidation and signaling

The intermediate stage of ferroptosis is characterised by the extent of lipid peroxidation and the activation of redox signalling that commits the cardiomyocytes to irreversible damage. Through the fenton reaction, elevated levels of labile Fe^2+ ^catalyse the generation of hydroxyl radicals, which start lipid oxidation by removing hydrogen atoms from bis-allylic sites in PUFAs (Cui and Ye, 2026[14]). Dox and Fe^3+ ^can combine to form a Dox-Fe^3+ ^complex, which further gets converted into a Dox-Fe^2+ ^complex. A superoxide radical O^2-^, and then a hydroxyl radical (OH• ) is created when the Dox-Fe^2+ ^complex combines with oxygen (Myers, 1998[50]). This stimulates a series of downstream events that lead to the build-up of lipid hydroperoxides. This process is further accelerated by enzymatic means, especially ACSL4, which selectively oxidizes PUFA-phospholipids and accelerates the ferroptosis process (Ding et al., 2023[16]). Cardiolipin and other PUFA-rich phospholipids are abundant in the heart and greatly increase vulnerability to this oxidative cascade (Zhang et al., 2025[88]).

Secondary reactive aldehydes, including malondialdehyde and 4-hydroxynonenal, are increased when cardiomyocytes are exposed to Dox (Luo et al., 1997[44]). These electrophilic molecules form covalent adducts with proteins, nucleic acids, and membrane constituents, resulting in extensive cellular damage.

### 4.3 Late stage: Cell death, tissue remodeling, and heart failure

**Figure 3:**
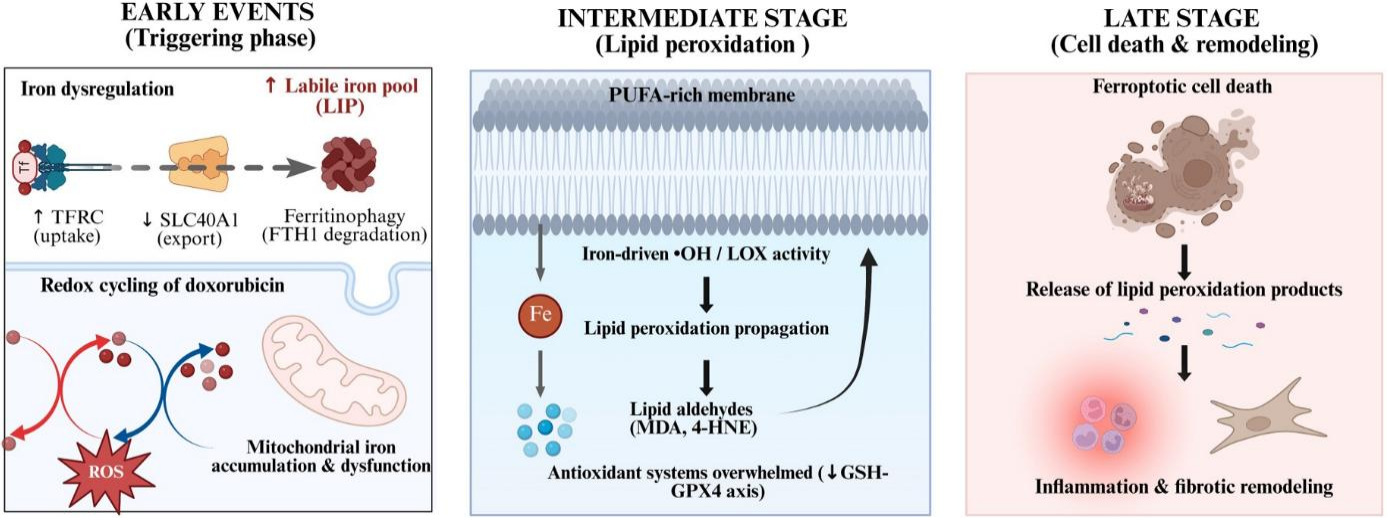
Different stages of toxicity and the role of ferroptosis in doxorubicin-induced cardiotoxicity. Doxorubicin promotes iron dysregulation and reactive oxygen species (ROS) generation, leading to lipid peroxidation, impaired antioxidant defenses, mitochondrial damage, and ferroptotic cell death in cardiomyocytes. The figure was created using BioRender software.

Ferroptosis is considered a key mediator of late-stage chronic cardiomyopathy, driven by excessive accumulation of lipid hydroperoxides in PUFA-rich membranes. This causes the generation of highly reactive electrophilic molecules that surpass the cellular glutathione-GPX4 system's detoxification capacity and lead to membrane damage (Forcina and Dixon, 2019[24]). This involves degradation of membrane-bound proteins, ion gradient disruption, and loss of plasma membrane integrity (Forcina and Dixon, 2019[24]). The typical ferroptotic shape of mitochondria, which reflects severe oxidative damage, includes condensed structure, increased membrane density, and a lack of cristae (Otasevic et al., 2021[54]). In contrast to apoptosis, nuclear integrity is mostly maintained, but cellular function is permanently impaired, which in later stages results in membrane rupture resembling necrosis (Ai et al., 2024[1]). This gradually progresses to a stage where myocardial tissue is remodelled by fibrotic tissue, and eventually cardiac dysfunction (Sun et al., 2026[66]). In an effort to make up for this dysfunctionality, the normal cardiomyocytes undergo hypertrophic alterations, which are often functionally inefficient and even contribute to a vicious circle of cellular stress (Sun et al., 2026[66]). Over time, these structural and functional changes result in progressive cardiac dysfunction, which clinically manifests as ventricular dilation, reduced ejection fraction, and eventually heart failure (Arrigoni et al., 2025[3]). In this manner, ferroptosis bridges cardiomyocyte structural damage to its functional impairment by acting as a driver of cellular pathology rather than just a cell death mechanism (Figure 3(Fig. 3)).

## 5. Role of Ferroptosis in Mitochondrial Damage

Dox shows high mitochondrial accumulation, especially in cardiomyocytes (Wallace et al., 2020[73]). It is also reported that Dox tends to accumulate in mitochondria at a concentration 100 times higher than in plasma (Wu et al., 2022[77]). Mitochondria are double-membraned intracellular organelles that function as the powerhouse of eukaryotic cells and are responsible for various cellular functions, including energy production, cellular metabolism, and redox signaling (Moura et al., 2025[49]). Their integrity is particularly essential for cardiomyocyte survival and function (Lazaropoulos and Elrod, 2022[38]). Cardiomyocytes, mitochondria-rich cells, which comprise about 70 % of the myocardial volume, contain mitochondria that occupy approximately one-third of the cellular space (Li et al., 2023[39]). Ferroptosis, a cellular mechanism that damages mitochondria, is a major cause of cellular redox imbalance between the oxidant and antioxidant systems and iron-dependent lipid peroxidation (Javadov, 2022[35]). Although the relationship between dysregulation of iron homeostasis and cardiovascular health was studied nearly thirty years ago, the exact mechanism responsible for this remained unexplored until the concept of an iron-dependent form of regulated cell death was proposed a decade ago (Fang et al., 2023[21]). The relationship between ferroptosis and mitochondrial dysfunction is highly dynamic and bidirectional (Li et al., 2026[41]). As mitochondria contribute to ferroptosis through the generation of ROS and regulation of iron metabolism, on the other hand, ferroptosis directly damages mitochondrial structure and function (Wang et al., 2021[74]). This interplay establishes a self-amplifying cycle that accelerates cardiomyocyte injury and functional decline (Fang et al., 2019[22]). Cardiotoxicity caused by Dox involves nitrosative and oxidative stress in cardiomyocytes, generating reactive nitrogen and oxygen species (Bhadra et al., 2025[6]; Songbo et al., 2019[64]). These species cause cell death through a variety of mechanisms, including disruption of normal mitochondrial function, an increase in NADPH oxidase, which causes ROS production, and iron overloading, which accumulates free hydroxyl radicals that cause mitochondrial damage through lipid peroxidation and ferrous ion accumulation, which in turn disrupts lipids in membranes and ferroptosis-induced cell death (Asensio-Lopez et al., 2017[5]; Bhadra et al., 2025[6]; Wang et al., 2021[74]). In the setting of Dox-induced cardiotoxicity, mitochondrial dysfunction, driven by oxidative stress, is one of the earliest and most prominent pathological features, and ferroptosis emerges as a critical mechanism that perpetuates oxidative stress-induced mitochondrial injury (Ye et al., 2024[84]).

### 5.1 Mitochondrial iron overload and redox imbalance

Being involved in a number of physiological processes, iron is considered to be an essential cellular element (Silva and Faustino, 2015[63]). However, if the concentration of this element reaches a toxic level, such as in the case of iron overload, it can even lead to dysfunction of the body's major organs (Gordan et al., 2018[27]). Ferroptosis, due to increased iron accumulation, has been well studied in the context of cardiotoxicity (Xie et al., 2022[80]).

A central event in ferroptosis-mediated mitochondrial damage is the dysregulation of intracellular iron homeostasis (Tayal et al., 2025[70]). Dox disrupts iron metabolism by interfering with the expression of iron regulatory proteins and thus promoting the release of iron from intracellular storage complexes, such as ferritin (Bhadra et al., 2025[6]; Yang et al., 2019[82]). Dox inhibits iron uptake by regulating iron-regulatory proteins such as IRP1 and IRP2, which regulate the expression of the transferrin receptor and ferritin genes. As Dox generates ROS, there is iron accumulation, which affects the activity of IRPs and leads to the accumulation of iron within the cells (Bhadra et al., 2025[6]). This leads to an increase in the LIP, a highly reactive form of iron that can participate in redox reactions (Ru et al., 2024[61]).

Excess iron within mitochondria catalyzes the fenton reaction, resulting in the production of highly reactive hydroxyl radicals (Ru et al., 2024[61]; Yu et al., 2021[87]). These radicals are highly reactive and non-specific, leading to widespread oxidation of mitochondrial macromolecules. mtDNA lacks the guarding histones and has limited repair capacity, making it susceptible to oxidative damage (Hanna et al., 2019[30]). Simultaneously, iron-induced oxidation of mitochondrial proteins disrupts mitochondrial metabolic activity, whereas elevated lipid oxidation impairs mitochondrial membrane integrity. Consequently, these effects result in a disruption in redox homeostasis, progressing the cell toward ferroptotic cell death (Yang and Stockwell, 2016[83]).

### 5.2 Lipid peroxidation of mitochondrial membranes

PUFAs, which contain multiple double bonds in their chemical structure, are highly prone to peroxidation by free radicals. Lipid peroxidation of mitochondrial phospholipids containing PUFAs is marked by the accumulation of iron, which enhances the fenton reaction and produces more ROS (Stockwell et al., 2017[65]). Cardiolipin, strategically located in the inner mitochondrial membrane, plays a crucial role in maintaining the function of the ETC and the integrity of the mitochondrial membrane (Paradies et al., 2019[55]). Therefore, peroxidation of cardiolipin during ferroptosis results in disruption of the mitochondrial membrane's structural organization, leading to loss of membrane integrity and increased permeability (Bucarey et al., 2026[7]). Lipid peroxidation alters membrane fluidity, triggers the formation of lipid pores, and further exacerbates mitochondrial dysfunction, indicating that the accumulation of lipid peroxides is not merely a consequence but a driving force of ferroptotic cell death (Mortensen et al., 2023[48]).

### 5.3 Impairment of antioxidant defense systems

The cellular defense against lipid peroxidation is primarily mediated by the glutathione (GSH)-dependent antioxidant system, with GPX4 serving as a key enzyme (W. Zhang et al., 2024[89]). Under typical conditions, the detoxification of lipid peroxides is supported by the GSH-GPX4 antioxidant pathway, thereby preserving mitochondrial integrity (Bucarey et al., 2026[7]). Ferroptosis, on the other hand, causes GSH depletion and GPX4 inactivation, leading to lipid peroxidation and worsening mitochondrial oxidative damage (Zhang et al., 2024[89]). GPX4 reduces lipid hydroperoxides to non-toxic lipid alcohols, thereby preventing membrane damage, while in Dox-treated cardiomyocytes, this protective system is severely compromised (Tadokoro et al., 2020[67]).

**Figure 4:**
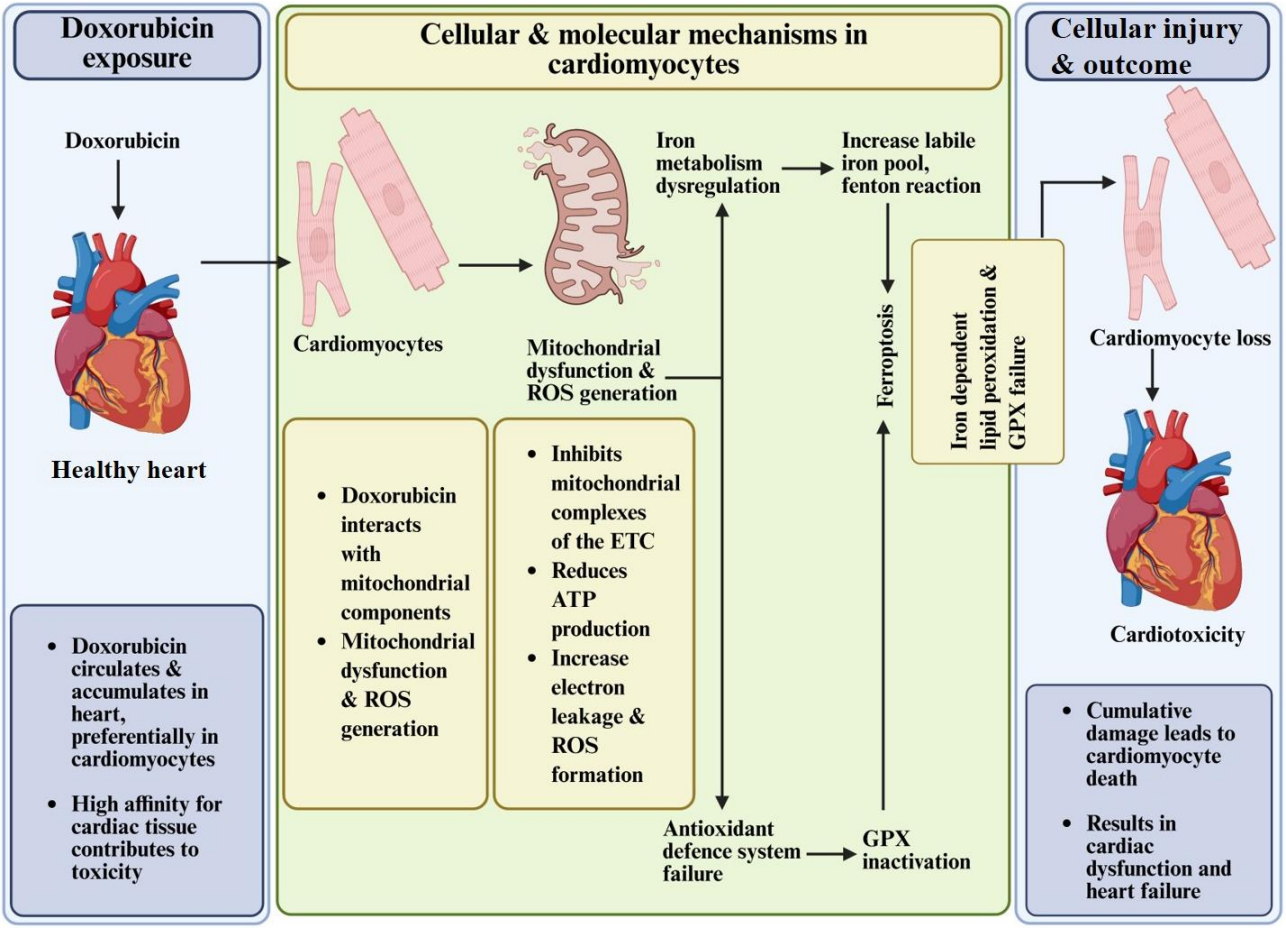
Pictorial representation of cellular and molecular mechanisms in doxorubicin-induced cardiotoxicity. The figure illustrates major processes, including oxidative stress, mitochondrial dysfunction, and dysregulated iron metabolism, contributing to cardiomyocyte damage. The figure was created using BioRender software.

Dox induces depletion of intracellular GSH levels by increasing oxidative stress and impairing synthesis. Simultaneously, it inhibits GPX4 activity either directly or indirectly through oxidative modifications (Wu et al., 2024[78]). The loss of GPX4 function leads to the accumulation of lipid hydroperoxides, which propagate lipid oxidation. The impaired regulation of the GPX4 antioxidant system further amplifies oxidative damage and thus mitochondrial membrane destabilization (Figure 4(Fig. 4)) (Tadokoro et al., 2020[67]; Wu et al., 2024[78]). At the same time, Dox decreases the cellular ability to detoxify lipid hydroperoxides by reducing intracellular glutathione levels and impairing GPX4 (system Xc-/GPX4 axis), a critical inhibitor of ferroptosis (Doroshow et al., 1980[20]). Tadokoro and his team reported significant downregulation of the GSH/GSSG ratio in Dox-challenged cardiomyocytes (Tadokoro et al., 2020[67]).

### 5.4 Disruption of the electron transport chain and alterations in mitochondrial morphology

ETC in the mitochondria is vital for cellular energy homeostasis (Nolfi-Donegan et al., 2020[52]). Dox interacts with ETC complexes, impairing electron transfer and increasing electron leakage, thereby enhancing ROS production and reducing oxidative phosphorylation efficiency (Huang et al., 2022[32]). Dox particularly localizes to the inner mitochondrial membrane through cardiolipin binding, and Dox undergoes redox cycling at complex I and III, which produces excess superoxide and leads to impaired electron flow through the ETC (Aryal and Rao, 2016[4]; Wallace et al., 2020[73]).

Increased mtROS generation by TCA cycle intermediates like succinate, malate, and fumarate accounts for the stimulation of mitochondrial glutaminolysis and precipitation of ferroptosis.

As a result, ATP production decreases, leading to an energy deficit in cardiac cells (Huang et al., 2022[32]). Given the high energy demand of cardiac tissue, this bioenergetic disturbance leads to functional changes, including impaired contractility and increased susceptibility to cell death (Wu et al., 2024[78]).

Distinct morphological alterations in mitochondria show characteristics of ferroptosis and provide important insights into its role in cardiotoxicity (Fang et al., 2023[21]). There is a significant shrinkage of mitochondria with an associated decrease in the number of cristae, representative of structural damage (Wu et al., 2024[78]). This structural damage disrupts the ETC, leading to functional impairment too (Fang et al., 2023[21]; Wu et al., 2024[78]).

### 5.5 Crosstalk/interactions with other cell death signaling

Ferroptosis works in a coordinated manner with other programmed cell death mechanisms, with mitochondria as a central mediator (Wu et al., 2023[79]). The structural and functional damage to mitochondria causes the release of cytochrome c, which initiates the process of apoptosis (Lu et al., 2026[43]; Wu et al., 2023[79]). Recent studies have also proposed a significant crosstalk between apoptosis and ferroptosis through the proapoptotic molecule PUMA (p53 upregulated modulator of apoptosis). Ferroptotic agents like erastin have been demonstrated to trigger endoplasmic reticulum stress, which further activates the PERK-eIF2α-ATF4-CHOP pathway and upregulates the expression of PUMA, rendering the cell susceptible to apoptosis.

These cells interactively undergo apoptosis and ferroptosis, thus exhibiting microscopic features of both (Lu et al., 2026[43]; Qiu et al., 2025[58]).

Similarly, ferroptosis works hand in hand with autophagy, being extensively described in studies related to cardiovascular diseases (Hu et al., 2025[31]). This process of ferritinophagy is also observed in Dox-induced cardiotoxicity, where it is mediated by the binding of the cytosolic autophagy receptor NCOA4 to FTH1, promoting the degradation of ferritin and iron accumulation, leading to ferroptosis (Wu et al., 2024[78]).

Necrotic pathways, particularly those involving mitochondrial permeability transition, may also intersect with ferroptosis under conditions of severe oxidative stress, serving lipid peroxidation and loss of membrane integrity (Christidi and Brunham, 2021[13]; Lu et al., 2026[43]). Although there is no specific study showing interactions between ferroptosis and necrosis, the evidence shows ferroptosis leads to membrane damage, ROS production, and lipid accumulation, and these phenotypes are similar to necrosis (Christidi and Brunham, 2021[13]). These connections to ferroptosis as a distinct feature imply the need to analyze multiple cell death signaling pathways.

## 6. Ferroptosis Position in the Cardiotoxicity Cascade

As ferroptosis is gaining attention as a target for Dox-induced cardiotoxicity, understanding its role as a driver and amplifier of signaling is crucial.

### 6.1 Early events/upstream initiators/pre-ferroptosis phase

As discussed above, Dox-induced cardiotoxicity is associated with oxidative stress and disrupted iron homeostasis, which are closely associated with activation of ferroptosis (Wu et al., 2024[78]). In the initial phase, the quinone moiety of Dox is responsible for ROS production and its interaction with mitochondrial ETC components (Linders et al., 2024[42]). This leads to excessive production of hydrogen peroxide, superoxide anions, and disrupts antioxidant defence systems, and dox also affects systemic and intracellular iron homeostasis, also shown to interfere with iron regulatory proteins and promote the release of iron from intracellular stores such as ferritin, thereby increasing lipid peroxidation (Wu et al., 2024[78]). Additionally, Dox-iron complexes further augments redox cycle, facilitating Fenton reactions that produce highly reactive hydroxyl radicals, which are responsible for damaging lipid membranes (Vitale et al., 2024[72]). Excessive ROS production and iron dysregulation create an oxidative intracellular environment, a precondition for ferroptosis initiation (Linders et al., 2024[42]). The LIP may stimulate enzymes like lipoxygenases or polyhydroxylases that control lipid peroxidation and oxygen balance. Importantly, these early molecular events promote structural and functional damage in cardiomyocytes, indicating that ferroptosis-related processes are initiated at an early stage in the Dox-induced cardiotoxicity signaling pathway. (Linders et al., 2024[42]; Vitale et al., 2024[72]).

### 6.2 Condition-dependent role of ferroptosis

Ferroptosis in cardiotoxicity is context-dependent, as various factors such as duration of exposure, dose, and the metabolic state of cardiomyocytes significantly influence the timing and extent of ferroptosis (Bhadra et al., 2025[6]). The exact position of ferroptosis in Dox-induced cellular death is determined, whether the drug has been administered for an acute duration or for chronic use. Dox, when administered at high doses even for an acute duration, generates ROS. It has been discussed above that any imbalance in redox homeostasis will lead to peroxidation of membrane phospholipids and disruption in the antioxidant defense mechanisms, resulting in iron accumulation that directly triggers ferroptosis. This fact puts this process as an early driver of cell death. On the other hand, when Dox is administered for a longer duration, even at a low dose, the induced ferroptosis will act as an amplifier for a series of molecular events progressing to cell death (Wang et al., 2025[75]). This cellular fate is also dependent on the status of the endogenous antioxidant mechanism, which decides the susceptibility as well as the extent of ferroptosis-induced damage (Feng et al., 2023[23]). Here, the ferroptosis acts as an intermediate component that directs oxidative stress and iron accumulation towards permanent cell damage. It can be, therefore, stated that factors associated with clinical conditions will tell about the position of ferroptosis in the cell death cascade.

## 7. Approaches Required to Conclude the Driver–Amplifier Debate

The final question that is raised by this review article is whether the process of ferroptosis is an initiator or an amplifier of the regulated cell death mechanism. It is a complex issue that is addressed by considering the complex nature of various cellular mechanisms encompassing the ferroptotic pathway. This involves the regulation of cellular and mitochondrial oxidative stress, the functioning of mitochondria, and other associated cell death signalling pathways. Hence, the approach required to reach any conclusion regarding the position of ferroptosis necessitates the development and study of a multidimensional framework of Dox-induced cardiotoxicity event.

### 7.1 Ferroptosis as a primary driver

There are studies indicating that ferroptosis acts not merely as one of the regulated cell death mechanisms involved in Dox-induced cardiac cell death but rather as a primary driver that initiates the whole cardiotoxicity (Fang et al., 2023[21]). Agents such as ferrostatin-1 and liproxstatin-1, which specifically inhibit lipid peroxidation and ferroptotic cell death, have been demonstrated to ameliorate drug-induced cardiotoxicity in both *in vitro* and *in vivo* models (Cha et al., 2025[9]; Fang et al., 2023[21]; Yu et al., 2026[85]). These studies suggest that ferroptosis plays a causative role as an early event in cell death.

Ferroptosis-induced iron accumulation and lipid peroxidation may be considered as instigating episodes, but they are not the final facilitators of Dox-induced cardiac cell death. A hallmark of ferroptosis is a reduction in GSH levels, accompanied by the inactivation of GPX4, which is responsible for lipid peroxidation (Zhang et al., 2024[89]). The resultant accumulation of lipid peroxidation products, such as malondialdehyde (MDA) and 4-hydroxynonenal (4-HNE), is typically observed before any notable signs of apoptosis or necrosis appear, supporting the early activation of ferroptotic pathways (Yan et al., 2021[81]). This finding is also corroborated by some genetic studies where overexpression of GPX4 or upregulation of system Xc⁻ (via SLC7A11) has been demonstrated to protect against Dox-induced cardiotoxicity (Zhang et al., 2024[91]). In this context, ferroptosis can be recognized as a key early determinant of cardiac cell death and a promising target for adjunct therapy with doxorubicin.

### 7.2 Ferroptosis as a downstream consequence

In contrast to the above evidence indicating early involvement of ferroptosis, other studies point to it as a downstream amplifier in the cell death signalling cascade. Dox-induced mitochondrial damage, including impaired oxidative phosphorylation and increased electron leakage, can independently generate ROS. Studies have shown an early ROS production and underlying mitochondrial dysfunction even before the upregulation of ferroptotic markers (Chen et al., 2024[10]). Moreover, classical markers of ferroptosis, such as lipid peroxidation and GPX4 depletion, are often seen after the progression of oxidative stress and mitochondrial damage. This sequence implies that ferroptosis may be secondary to initial cellular insults and may serve to exacerbate rather than initiate damage (Tang et al., 2021[68]).

In this context, ferroptosis can be viewed as a feed-forward mechanism that amplifies pre-existing oxidative and metabolic stress. Once triggered, ferroptotic processes further increase lipid peroxidation and ROS production, thereby accelerating cellular dysfunction and death. This perspective is consistent with the observation that ferroptosis inhibitors are often more effective when administered during later stages of injury, suggesting a role in limiting damage progression rather than preventing its initiation (Chen et al., 2024[10]; Tang et al., 2021[68]). These observations propose a concept of the ferroptotic vicious circle, where the cardiac cell damage triggers ferroptosis, which further amplifies the cardiac injury, ultimately leading to cardiac cell death.

## 8. Conclusion

Ferroptosis is gaining attention as a regulated cell death pathway that outlines the cellular as well as molecular pathological aspects of Dox-induced cardiotoxicity. Reviewing this process indicates that the heart doesn't just behave as a passive target of the drug-induced toxicity but as an organized system that is predisposed to ferroptotic cell death because of its dynamic iron metabolism, high mitochondrial density, and highly enriched polyunsaturated fatty acid chains within mitochondrial and cellular membranes. Dox exploits cardiomyocyte physiological features and initiates a range of interdependent processes that culminate in ferroptotic cell death. Addressing the central theme of this review, whether ferroptosis acts as an initiator or an amplifier for Dox-induced cardiotoxicity, does not present an explicit answer. Based on the available literature, it can be concluded that ferroptosis is a context-dependent, dynamic process that transitions from a driver to a dominant downstream amplifier during the progression of cardiotoxicity.

## Notes

Nisha Sharma and Umashanker Navik (Department of Pharmacology, Central University of Punjab, Bathinda, Punjab 151401, India; E-mail: usnavik@gmail.com & uma.shanker@cup.edu.in) contributed equally as corresponding author.

## Declaration

### Conflict of interest

The authors declare that they have no conflict of interest.

### Funding

No funding was received.

### Data availability

Not applicable.

### Ethics approval and consent to participate

Not applicable.

### Artificial Intelligence (AI) - assisted technology

The authors used Grammarly software to improve the readability and language of the manuscript. After using this tool, the authors reviewed and edited the content as needed and take full responsibility for the content of the published article.

### Author contribution

Yogender Goswami: Data Analysis, Writing the Draft, Original Draft Preparation; Nisha Sharma and Umashanker Navik: Writing - Reviewing and Editing, Supervision

## Figures and Tables

**Figure 1:**
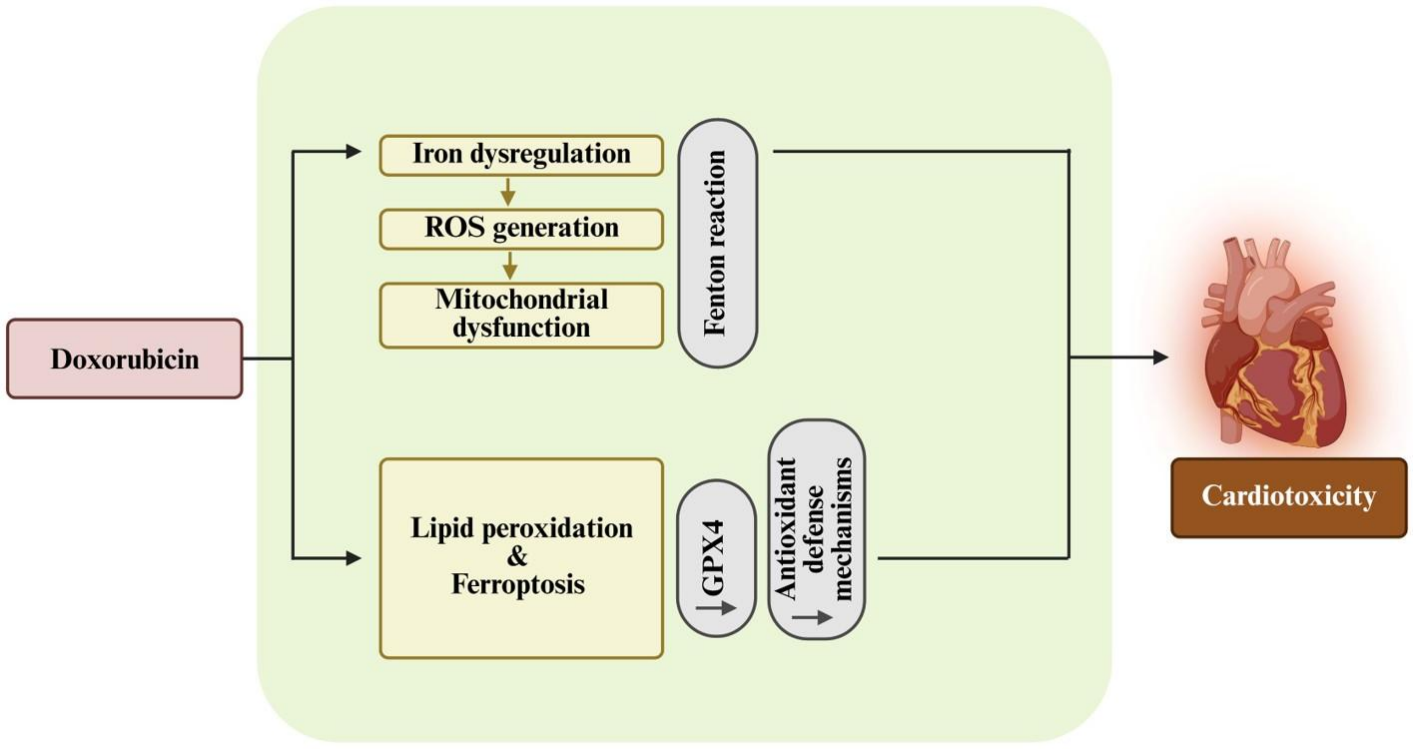
Graphical abstract

## References

[1] Ai Y, Meng Y, Yan B, Zhou Q, Wang X (2024). The biochemical pathways of apoptotic, necroptotic, pyroptotic, and ferroptotic cell death. Molecular cell.

[2] Arosio P, Elia L, Poli M (2017). Ferritin, cellular iron storage and regulation. IUBMB life.

[3] Arrigoni R, Jirillo E, Caiati C (2025). Pathophysiology of doxorubicin-mediated cardiotoxicity. Toxics.

[4] Aryal B, Rao VA (2016). Deficiency in Cardiolipin Reduces Doxorubicin-Induced Oxidative Stress and Mitochondrial Damage in Human B-Lymphocytes. PLoS One.

[5] Asensio-Lopez MC, Soler F, Pascual-Figal D, Fernandez-Belda F, Lax A (2017). Doxorubicin-induced oxidative stress: The protective effect of nicorandil on HL-1 cardiomyocytes. PLoS One.

[6] Bhadra P, Yadav P, Kaur S, Hanumantharayudu PT, Arunachalam S (2025). The role of ferroptosis in doxorubicin-induced cardiotoxicity–An update. Life Sciences.

[7] Bucarey JL, Casas M, Espinosa A (2026). Mitochondrial Iron Handling and Lipid Peroxidation as Drivers of Ferroptosis. Int J Mol Sci.

[8] Cappetta D, De Angelis A, Sapio L, Prezioso L, Illiano M, Quaini F (2017). Oxidative stress and cellular response to doxorubicin: a common factor in the complex milieu of anthracycline cardiotoxicity. Oxidative medicine and cellular longevity.

[9] Cha YJ, Jeon SB, Lee CJ, Kim HJ, Lee SH, Kim H (2025). Limitations of Ferroptosis Inhibitors on the Doxorubicin-Induced Cardiotoxicity. Antioxidants (Basel).

[10] Chen F, Kang R, Tang D, Liu J (2024). Ferroptosis: principles and significance in health and disease. J Hematol Oncol.

[11] Chen X, Yu C, Kang R, Tang D (2020). Iron metabolism in ferroptosis. Frontiers in cell and developmental biology.

[12] Cho S, Tam E, Nguyen K, Lei Y, Fillebeen C, Pantopoulos K (2025). ω-6 PUFA-enriched membrane phospholipid composition of cardiomyocytes increases the susceptibility to iron-induced ferroptosis and inflammation. Apoptosis.

[13] Christidi E, Brunham LR (2021). Regulated cell death pathways in doxorubicin-induced cardiotoxicity. Cell Death Dis.

[14] Cui S, Ye J (2026). Ferroptosis: The Demise of Cells Through Phospholipid Peroxidation. Advanced Science.

[15] Deo M, Weinberg SH, Boyle PM (2017). Calcium dynamics and cardiac arrhythmia. Clinical Medicine Insights: Cardiology.

[16] Ding K, Liu C, Li L, Yang M, Jiang N, Luo S (2023). Acyl-CoA synthase ACSL4: an essential target in ferroptosis and fatty acid metabolism. Chinese medical journal.

[17] Dixon SJ, Lemberg KM, Lamprecht MR, Skouta R, Zaitsev EM, Gleason CE (2012). Ferroptosis: an iron-dependent form of nonapoptotic cell death. Cell.

[18] Dixon SJ, Olzmann JA (2024). The cell biology of ferroptosis. Nature reviews Molecular cell biology.

[19] Donovan A, Lima CA, Pinkus JL, Pinkus GS, Zon LI, Robine S (2005). The iron exporter ferroportin/Slc40a1 is essential for iron homeostasis. Cell metabolism.

[20] Doroshow JH, Locker GY, Myers C (1980). Enzymatic defenses of the mouse heart against reactive oxygen metabolites: alterations produced by doxorubicin. The Journal of clinical investigation.

[21] Fang X, Ardehali H, Min J, Wang F (2023). The molecular and metabolic landscape of iron and ferroptosis in cardiovascular disease. Nature Reviews Cardiology.

[22] Fang X, Wang H, Han D, Xie E, Yang X, Wei J (2019). Ferroptosis as a target for protection against cardiomyopathy. Proc Natl Acad Sci U S A.

[23] Feng S, Tang D, Wang Y, Li X, Bao H, Tang C (2023). The mechanism of ferroptosis and its related diseases. Mol Biomed.

[24] Forcina GC, Dixon SJ (2019). GPX4 at the crossroads of lipid homeostasis and ferroptosis. Proteomics.

[25] Gammella E, Buratti P, Cairo G, Recalcati S (2017). The transferrin receptor: the cellular iron gate. Metallomics.

[26] Gammella E, Maccarinelli F, Buratti P, Recalcati S, Cairo G (2014). The role of iron in anthracycline cardiotoxicity. Frontiers in pharmacology.

[27] Gordan R, Wongjaikam S, Gwathmey JK, Chattipakorn N, Chattipakorn SC, Xie LH (2018). Involvement of cytosolic and mitochondrial iron in iron overload cardiomyopathy: an update. Heart Fail Rev.

[28] Green PS, Leeuwenburgh C (2002). Mitochondrial dysfunction is an early indicator of doxorubicin-induced apoptosis. Biochimica et Biophysica Acta (BBA)-Molecular Basis of Disease.

[29] Han H, Luo T, Liu C, Ou W, Zhang Y, Zhang Z (2026). Vericiguat as a Novel Ferroptosis Inhibitor Alleviates Doxorubicin‐Induced Cardiotoxicity. Basic & Clinical Pharmacology & Toxicology.

[30] Hanna R, Crowther JM, Bulsara PA, Wang X, Moore DJ, Birch-Machin MA (2019). Optimised detection of mitochondrial DNA strand breaks. Mitochondrion.

[31] Hu C, Gao S, Li X, Yang K, Cheng Y, Guo W (2025). Crosstalk of autophagy and ferroptosis in cardiovascular diseases: from pathophysiology to novel therapy. Redox Biol.

[32] Huang J, Wu R, Chen L, Yang Z, Yan D, Li M (2022). Understanding Anthracycline Cardiotoxicity From Mitochondrial Aspect. Front Pharmacol.

[33] Ichikawa Y, Ghanefar M, Bayeva M, Wu R, Khechaduri A, Prasad SVN (2014). Cardiotoxicity of doxorubicin is mediated through mitochondrial iron accumulation. The Journal of clinical investigation.

[34] Jain D (2000). Cardiotoxicity of doxorubicin and other anthracycline derivatives. Journal of Nuclear Cardiology.

[35] Javadov S (2022). Mitochondria and ferroptosis. Curr Opin Physiol.

[36] Kitakata H, Endo J, Matsushima H, Yamamoto S, Ikura H, Hirai A (2021). MITOL/MARCH5 determines the susceptibility of cardiomyocytes to doxorubicin-induced ferroptosis by regulating GSH homeostasis. Journal of molecular and cellular cardiology.

[37] Kumar A, Tiwari S (2025). Doxorubicin-induced Acute Cardiotoxicity and Left Ventricular Failure After Cytoreductive Surgery and Hyperthermic Intraperitoneal Chemotherapy. Journal of Onco-Anaesthesiology and Perioperative Medicine.

[38] Lazaropoulos MP, Elrod JW (2022). Mitochondria in Pathological Cardiac Remodeling. Curr Opin Physiol.

[39] Li A, Shami GJ, Griffiths L, Lal S, Irving H, Braet F (2023). Giant mitochondria in cardiomyocytes: cellular architecture in health and disease. Basic Res Cardiol.

[40] Li FJ, Long HZ, Zhou ZW, Luo HY, Xu SG, Gao LC (2022). System Xc−/GSH/GPX4 axis: An important antioxidant system for the ferroptosis in drug-resistant solid tumor therapy. Frontiers in pharmacology.

[41] Li M, Zhang Y, Hu Y, Qin Y, Zheng Y, Lv S (2026). Mitochondrial homeostasis meets novel programmed cell death: crosstalk mechanisms underlying cardiovascular diseases progression. Cell Communication and Signaling.

[42] Linders AN, Dias IB, López Fernández T, Tocchetti CG, Bomer N, Van der Meer P (2024). A review of the pathophysiological mechanisms of doxorubicin-induced cardiotoxicity and aging. NPJ Aging.

[43] Lu W, Deng Y, Liu M, Hu Y, Yang K, Wang B (2026). Regulation of apoptosis, ferroptosis, and pyroptosis mediated by acetylation. Cell Death Discov.

[44] Luo X, Evrovsky Y, Cole D, Trines J, Benson LN, Lehotay DC (1997). Doxorubicin-induced acute changes in cytotoxic aldehydes, antioxidant status and cardiac function in the rat. Biochimica et Biophysica Acta (BBA)-Molecular Basis of Disease.

[45] Lyu YL, Kerrigan JE, Lin CP, Azarova AM, Tsai YC, Ban Y (2007). Topoisomerase IIβ–mediated DNA double-strand breaks: implications in doxorubicin cardiotoxicity and prevention by dexrazoxane. Cancer research.

[46] Minotti G, Menna P, Salvatorelli E, Cairo G, Gianni L (2004). Anthracyclines: molecular advances and pharmacologic developments in antitumor activity and cardiotoxicity. Pharmacological reviews.

[47] Montaigne D, Marechal X, Preau S, Baccouch R, Modine T, Fayad G (2011). Doxorubicin induces mitochondrial permeability transition and contractile dysfunction in the human myocardium. Mitochondrion.

[48] Mortensen MS, Ruiz J, Watts JL (2023). Polyunsaturated fatty acids drive lipid peroxidation during ferroptosis. Cells.

[49] Moura JP, Oliveira PJ, Urbano AM (2025). Mitochondria: An overview of their origin, genome, architecture, and dynamics. Biochim Biophys Acta Mol Basis Dis.

[50] Myers C (1998). The role of iron in doxorubicin-induced cardiomyopathy. Seminars in oncology.

[51] Nemeth E, Ganz T (2021). Hepcidin-ferroportin interaction controls systemic iron homeostasis. International journal of molecular sciences.

[52] Nolfi-Donegan D, Braganza A, Shiva S (2020). Mitochondrial electron transport chain: Oxidative phosphorylation, oxidant production, and methods of measurement. Redox Biol.

[53] Octavia Y, Tocchetti CG, Gabrielson KL, Janssens S, Crijns HJ, Moens AL (2012). Doxorubicin-induced cardiomyopathy: from molecular mechanisms to therapeutic strategies. Journal of molecular and cellular cardiology.

[54] Otasevic V, Vucetic M, Grigorov I, Martinovic V, Stancic A (2021). Ferroptosis in different pathological contexts seen through the eyes of mitochondria. Oxidative medicine and cellular longevity.

[55] Paradies G, Paradies V, Ruggiero FM, Petrosillo G (2019). Role of Cardiolipin in Mitochondrial Function and Dynamics in Health and Disease: Molecular and Pharmacological Aspects. Cells.

[56] Ping Z, Fangfang T, Yuliang Z, Xinyong C, Lang H, Fan H (2023). Oxidative Stress and Pyroptosis in Doxorubicin‐Induced Heart Failure and Atrial Fibrillation. Oxidative Medicine and Cellular Longevity.

[57] Qin Y, Guo T, Wang Z, Zhao Y (2021). The role of iron in doxorubicin-induced cardiotoxicity: recent advances and implication for drug delivery. Journal of Materials Chemistry B.

[58] Qiu Y, Huther JA, Wank B, Rath A, Tykwe R, Aldrovandi M (2025). Interplay of ferroptotic and apoptotic cell death and its modulation by BH3-mimetics. Cell Death Differ.

[59] Rawat PS, Jaiswal A, Khurana A, Bhatti JS, Navik U (2021). Doxorubicin-induced cardiotoxicity: An update on the molecular mechanism and novel therapeutic strategies for effective management. Biomedicine & Pharmacotherapy.

[60] Rivankar S (2014). An overview of doxorubicin formulations in cancer therapy. Journal of cancer research and therapeutics.

[61] Ru Q, Li Y, Chen L, Wu Y, Min J, Wang F (2024). Iron homeostasis and ferroptosis in human diseases: mechanisms and therapeutic prospects. Signal Transduct Target Ther.

[62] Shinlapawittayatorn K, Chattipakorn SC, Chattipakorn N (2022). The effects of doxorubicin on cardiac calcium homeostasis and contractile function. Journal of cardiology.

[63] Silva B, Faustino P (2015). An overview of molecular basis of iron metabolism regulation and the associated pathologies. Biochimica et Biophysica Acta (BBA) - Molecular Basis of Disease.

[64] Songbo M, Lang H, Xinyong C, Bin X, Ping Z, Liang S (2019). Oxidative stress injury in doxorubicin-induced cardiotoxicity. Toxicology letters.

[65] Stockwell BR, Friedmann Angeli JP, Bayir H, Bush AI, Conrad M, Dixon SJ (2017). Ferroptosis: A Regulated Cell Death Nexus Linking Metabolism, Redox Biology, and Disease. Cell.

[66] Sun Y, Xiao L, Chen L, Wang X (2026). Doxorubicin-Induced cardiac remodeling: mechanisms and mitigation strategies. Cardiovascular Drugs and Therapy.

[67] Tadokoro T, Ikeda M, Ide T, Deguchi H, Ikeda S, Okabe K (2020). Mitochondria-dependent ferroptosis plays a pivotal role in doxorubicin cardiotoxicity. JCI insight.

[68] Tang D, Chen X, Kang R, Kroemer G (2021). Ferroptosis: molecular mechanisms and health implications. Cell Research.

[69] Tang L, Zhang Y, Qian Z, Shen X (2000). The mechanism of Fe2+-initiated lipid peroxidation in liposomes: the dual function of ferrous ions, the roles of the pre-existing lipid peroxides and the lipid peroxyl radical. Biochemical Journal.

[70] Tayal A, Kaur J, Sadeghi P, Maitta RW (2025). Molecular Mechanisms of Iron Metabolism and Overload. Biomedicines.

[71] Vejpongsa P, Yeh E (2014). Topoisomerase 2β: a promising molecular target for primary prevention of anthracycline-induced cardiotoxicity. Clinical Pharmacology & Therapeutics.

[72] Vitale R, Marzocco S, Popolo A (2024). Role of Oxidative Stress and Inflammation in Doxorubicin-Induced Cardiotoxicity: A Brief Account. Int J Mol Sci.

[73] Wallace KB, Sardão VA, Oliveira PJ (2020). Mitochondrial determinants of doxorubicin-induced cardiomyopathy. Circulation research.

[74] Wang AJ, Zhang J, Xiao M, Wang S, Wang BJ, Guo Y (2021). Molecular mechanisms of doxorubicin-induced cardiotoxicity: novel roles of sirtuin 1-mediated signaling pathways. Cell Mol Life Sci.

[75] Wang B, Wang J, Liu C, Li C, Meng T, Chen J (2025). Ferroptosis: Latest evidence and perspectives on plant-derived natural active compounds mitigating doxorubicin-induced cardiotoxicity. J Appl Toxicol.

[76] Wang H, Liu C, Zhao Y, Gao G (2020). Mitochondria regulation in ferroptosis. European journal of cell biology.

[77] Wu BB, Leung KT, Poon EN (2022). Mitochondrial-Targeted Therapy for Doxorubicin-Induced Cardiotoxicity. Int J Mol Sci.

[78] Wu L, Zhang Y, Wang G, Ren J (2024). Molecular mechanisms and therapeutic targeting of ferroptosis in doxorubicin-induced cardiotoxicity. Basic to Translational Science.

[79] Wu P, Zhang X, Duan D, Zhao L (2023). Organelle-Specific Mechanisms in Crosstalk between Apoptosis and Ferroptosis. Oxid Med Cell Longev.

[80] Xie LH, Fefelova N, Pamarthi SH, Gwathmey JK (2022). Molecular Mechanisms of Ferroptosis and Relevance to Cardiovascular Disease. Cells.

[81] Yan HF, Zou T, Tuo QZ, Xu S, Li H, Belaidi AA (2021). Ferroptosis: mechanisms and links with diseases. Signal Transduction and Targeted Therapy.

[82] Yang R, Li Y, Wang X, Yan J, Pan D, Xu Y (2019). Doxorubicin loaded ferritin nanoparticles for ferroptosis enhanced targeted killing of cancer cells. RSC Adv.

[83] Yang WS, Stockwell BR (2016). Ferroptosis: Death by Lipid Peroxidation. Trends Cell Biol.

[84] Ye H, Wu L, Liu Y (2024). Iron metabolism in doxorubicin-induced cardiotoxicity: From mechanisms to therapies. Int J Biochem Cell Biol.

[85] Yu S, Pang Z, Fang H, Liu C (2026). Ferroptosis in cardiovascular diseases: molecular mechanisms and a novel therapeutic target. Molecular Biomedicine.

[86] Yu X, Ruan Y, Huang X, Dou L, Lan M, Cui J (2020). Dexrazoxane ameliorates doxorubicin-induced cardiotoxicity by inhibiting both apoptosis and necroptosis in cardiomyocytes. Biochemical and biophysical research communications.

[87] Yu Y, Yan Y, Niu F, Wang Y, Chen X, Su G (2021). Ferroptosis: a cell death connecting oxidative stress, inflammation and cardiovascular diseases. Cell Death Discovery.

[88] Zhang H, Yu F, Tian Z, Jia D (2025). Cardiolipin remodeling in cardiovascular diseases: implication for mitochondrial dysfunction. Acta Physiologica.

[89] Zhang W, Liu Y, Liao Y, Zhu C, Zou Z (2024). GPX4, ferroptosis, and diseases. Biomed Pharmacother.

[90] Zhang Y, Chen Y, Zhang M, Tang Y, Xie Y, Huang X (2014). Doxorubicin induces sarcoplasmic reticulum calcium regulation dysfunction via the decrease of SERCA2 and phospholamban expressions in rats. Cell biochemistry and biophysics.

[91] Zhang Y, Zou L, Li X, Guo L, Hu B, Ye H (2024). SLC40A1 in iron metabolism, ferroptosis, and disease: a review. WIREs mechanisms of disease.

[92] Zhao L, Zhang B (2017). Doxorubicin induces cardiotoxicity through upregulation of death receptors mediated apoptosis in cardiomyocytes. Scientific reports.

[93] Zhou YJ, Duan DQ, Lu LQ, Tang LJ, Zhang XJ, Luo XJ (2022). The SPATA2/CYLD pathway contributes to doxorubicin-induced cardiomyocyte ferroptosis via enhancing ferritinophagy. Chemico-biological Interactions.

